# Identification and Expression Analysis of Cytokinin Metabolic Genes in Soybean under Normal and Drought Conditions in Relation to Cytokinin Levels

**DOI:** 10.1371/journal.pone.0042411

**Published:** 2012-08-10

**Authors:** Dung Tien Le, Rie Nishiyama, Yasuko Watanabe, Radomira Vankova, Maho Tanaka, Motoaki Seki, Le Huy Ham, Kazuko Yamaguchi-Shinozaki, Kazuo Shinozaki, Lam-Son Phan Tran

**Affiliations:** 1 Signaling Pathway Research Unit, RIKEN Plant Science Center, Yokohama, Kanagawa, Japan; 2 National Key Laboratory of Plant Cell Biotechnology and Agricultural Genetics Institute, Vietnamese Academy of Agricultural Science, Hanoi, Vietnam; 3 Laboratory of Hormonal Regulations in Plants, Institute of Experimental Botany of the Academy of Sciences of the Czech Republic, Prague, Czech Republic; 4 Plant Genomic Network Research Team, RIKEN Plant Science Center, Yokohama, Kanagawa, Japan; 5 Japan International Research Center for Agricultural Sciences, Tsukuba, Ibaraki, Japan; 6 Gene Discovery Research Group, RIKEN Plant Science Center, Yokohama, Kanagawa, Japan; Kyushu Institute of Technology, Japan

## Abstract

Cytokinins (CKs) mediate cellular responses to drought stress and targeted control of CK metabolism can be used to develop drought-tolerant plants. Aiming to manipulate CK levels to improve drought tolerance of soybean cultivars through genetic engineering of CK metabolic genes, we surveyed the soybean genome and identified 14 CK biosynthetic (isopentenyltransferase, *GmIPT*) and 17 CK degradative (CK dehydrogenase, *GmCKX*) genes. Comparative analyses of GmIPTs and GmCKXs with *Arabidopsis* counterparts revealed their similar architecture. The average numbers of abiotic stress-inducible *cis*-elements per promoter were 0.4 and 1.2 for *GmIPT* and *GmCKX* genes, respectively, suggesting that upregulation of *GmCKX*s, thereby reduction of CK levels, maybe the major events under abiotic stresses. Indeed, the expression of 12 *GmCKX* genes was upregulated by dehydration in R2 roots. Overall, the expressions of soybean CK metabolic genes in various tissues at various stages were highly responsive to drought. CK contents in various organs at the reproductive (R2) stage were also determined under well-watered and drought stress conditions. Although tRNA-type *GmIPT* genes were highly expressed in soybean, *cis*-zeatin and its derivatives were found at low concentrations. Moreover, reduction of total CK content in R2 leaves under drought was attributable to the decrease in dihydrozeatin levels, suggesting a role of this molecule in regulating soybean's responses to drought stress. Our systematic analysis of the *GmIPT* and *GmCKX* families has provided an insight into CK metabolism in soybean under drought stress and a solid foundation for in-depth characterization and future development of improved drought-tolerant soybean cultivars by manipulation of CK levels via biotechnological approach.

## Introduction

Soybean (*Glycine max* L.), which is one of the major legume crops native to East Asia, provides an abundant source of oil and protein-rich food for both human and animal consumption. The growth and productivity of soybean are adversely affected by a number of environmental stresses [1], [2]. Among the adverse environmental factors commonly encountered by soybean, drought is considered the harshest, affecting all stages of plant growth and development. Drought stress typically results in significant yield losses and a reduction of seed quality for soybean [2], [3].

Generally, in response to drought stress, plants activate a wide range of defense mechanisms that function to increase tolerance to water limiting conditions [4]. The early events of a plant's adaptation to drought stress are the stress signal perception and subsequent signal transduction, leading to the activation of various physiological and metabolic responses [4]–[9]. In *Arabidopsis*, it has been reported that the signaling processes activated under water limiting conditions involve the conversion of stress signal perception to stress-responsive gene expression. The cytokinin (CK)-related two-component system (TCS), which consists of CK receptor histidine kinases (AHKs), His-containing phosphotransferases (AHPs) and response regulators (ARRs), function as molecular switches during stress responses. The utilization of CK receptor mutants as a central tool to study CK functions has led to the suggestion that CKs might mediate osmotic stress responses [5], [10]. Recent analyses of CK-deficient plants have demonstrated that CKs may act as negative regulators through CK signaling in response to drought and salt stresses [11]–[13].

CKs are produced in large quantities in proliferating tissues, such as root and shoot apical meristems, young leaves and immature seeds [14]. In *Arabidopsis*, the rate-limiting step of CK biosynthesis is catalyzed by the isopentenyltransferases (IPTs), which consist of ATP/ADP IPTs and tRNA IPTs. Studies on the biosynthetic pathways for these compounds have clarified that the ATP/ADP IPTs control the biosynthesis of isopentenyladenine (iP)- and *trans*-zeatin (*t*Z)-type CKs, whereas tRNA IPTs are responsible for the synthesis of *cis*-zeatin (*c*Z)-type CKs [14], [15]. On the other hand, CK degradation is catalyzed by the CK dehydrogenases (CKXs), which have distinct biochemical characteristics. For instance, in *Arabidopsis*, the AtCKX1 and AtCKX3 and AtCKX2 and AtCKX4 pairs possess similar function and substrate specificity as demonstrated by gain-of-function studies [16], [17]. Functional analyses of IPTs, CKXs and CK-related TCS members in *Arabidopsis* using both gain- and loss-of function approaches, have suggested that CKs control many biological processes, such as development, growth and cell division, in addition to responses to environmental stimuli [18]. CKs have been shown to negatively regulate root growth but positively regulate shoot growth in both vegetative and reproductive stages [16], [19], [20]; however, excessive overproduction of CKs above a threshold may cause stunted plant growth and abnormal tissue development [21]–[25]. Drought stress accelerates leaf senescence, which is associated with a decrease in CK content and suppression of CK signaling [26]–[29]. Strong lines of evidence have indicated that appropriate manipulation of CK levels may enhance tolerance to drought stress [30]–[32]. An overproduction of CKs during plant maturation, just prior to the onset of senescence, significantly increased drought tolerance with minimal yield loss due to a delay of drought-induced senescence associated with a pre-programmed increase in CK levels [33]–[35]. On the other hand, reduction of CK levels by the overexpression of *CKX* genes in roots promotes primary root elongation and root branching, resulting in an increase in root biomass that subsequently improves drought tolerance of transgenic plants [12].

Taking into account the importance of CKs and CK signaling in the regulation of stress tolerance, which provides multiple biotechnological strategies for agronomy, we have previously identified and characterized expression profiles of each TCS member in soybean seedlings under dehydration stress [36], [37]. In this report, we have identified and systematically characterized all of the IPT and CKX encoding genes in soybean. We have found that a large number of putative *GmIPT* and *GmCKX* genes may be the result of genome duplication. Since there is a wealth of structural and functional information for *Arabidopsis* IPTs and CKXs, we performed sequence analyses and phylogenetic relationship studies of IPTs and CKXs of soybean and *Arabidopsis* to classify the functions of GmIPT and GmCKX proteins in CK metabolism based on their sequence architecture. To clarify the regulation of CK metabolism in soybean during normal growth and drought stress, we have analyzed the expression patterns of *GmIPT* and *GmCKX* genes under normal and drought stress conditions in a tissue-specific fashion. Expression profiles of *GmIPT* and *GmCKX* genes were examined in various tissues of both dehydrated young seedlings and soil-dried plants at vegetative and flowering stages. Additionally, we have investigated the correlation between the drought stress-dependent alterations of CK metabolic gene expression and CK biosynthesis by determining the endogenous CK levels in soybean drought-treated leaf tissue.

## Materials and Methods

### Identification and annotation of the soybean *GmIPT* and *GmCKX* genes and in silico analyses

*Arabidopsis* IPTs and CKXs were applied as seed sequences to identify the GmIPT and GmCKX proteins in soybean (Glyma 1.0 version) using reciprocal blast as previously described [37]. Proteins, whose encoding sequences containing start and stop codons, were selected for further analysis. Selected protein sequences were searched against the PFAM database to confirm the presence of domain signatures. Protein sequence alignments were performed with a gap open penalty of 10 and gap extension penalty of 0.2 using ClustalW implemented in MEGA software [38], [39]. Unrooted phylogenetic trees were constructed using the neighbor-joining method. The confidence level of monophyletic groups was estimated using a bootstrap analysis of 10,000 replicates. Only bootstrap values higher than 40% are displayed next to the branch nodes.

Tandem duplicates were defined as those genes located within 20 loci from each other. Segmental duplications were identified by synteny analysis using an online tool (http://chibba.agtec.uga.edu/duplication/) [40].

To identify *cis*-regulatory motifs in the promoter regions of *GmIPT* and *GmCKX* genes, previously reported abiotic-stress related *cis*-elements[4] were used to search against the 1,000-bp upstream sequence of the transcriptional start site of each *GmIPT* or *GmCKX* gene using MEGA 4 software [39].

### Plant growth, dehydration and drought treatments and tissue collections

Growth and dehydration treatment of young soybean seedlings were performed as previously described [41]. Briefly, 12-day-old plants were removed from soil and roots were gently washed to remove soil. Plants were subsequently transferred onto filter paper and allowed to dry for 2 h and 10 h under 60% relative humidity, 25^°^C and 10 μmole m^−2^s^−1^ photon flux. For drought treatment, soybean plants (cv. Williams 82) were grown in pots (3 plants per 6-liter pot) containing Supermix (Supermix A, Sakata, Japan). Water was given to each pot once a day under greenhouse conditions (continuous 30°C temperature, photoperiod of 12 h/12 h, 80 μmol m^−2^ s^−1^ photon flux density and 50% relative humidity). Soybean plants at V6 stage (28 days after sowing, containing 7 trifoliate leaves) were withheld from watering to initiate the drought treatment. Water was provided to the well-watered control plants to maintain the volumetric soil moisture content (SMC) at 40–45%. At the sixth day of water withholding, where the SMC was below 5% and the soybean plants contained 7 fully open trifoliate leaves and a half-open 8^th^ trifoliate leaf (Figure S1A), soybean leaves were separately collected from each trifoliate leaf. The 3^rd^, 5^th^ and 7^th^ trifoliate leaves (counted from the bottom-up) were used for determination of the stress severity by measuring leaf relative water content (Figure S1B and S1C). At the same time, trifoliate leaves 4^th^, 6^th^ and 8^th^ were quickly frozen in liquid nitrogen and stored at −80^o^C for the isolation of RNA for qRT-PCR. All of the samples were collected in four biological replicates.

To collect the root and leaf tissue samples at reproductive stages (R2), the soybean plants were allowed to grow in a semi-hydroponic manner. The plants were grown in the pots as described above and the roots were allowed to outgrow through the soil layers to reach the water tray as seen in Figure S2. The hydroponic parts of auxiliary roots were cut while remained underwater. For dehydration treatment, the roots were removed from the water and kept on filter paper and allowed to dry at room temperature for 5 h. Control water-treated and dried roots were then collected and quickly frozen in liquid nitrogen and stored at −80^o^C until further use. All of the samples were collected in three biological replicates. Fully open flowers were collected during the R1 to R2 period in three biological replicates. Full pods were collected in three biological replicates during the R4 stage, of which all of the collected pods ranged from 10 to 20 mm in length. R5 seeds were collected during the R5 stage when the seeds were approximately 3 mm in length.

For the collection of well-watered and drought-treated leaves at the R2 reproductive stage, we measured the chlorophyll content of the 3^rd^ trifoliate leaves (counting down from the growing shoots) and marked the leaves with similar chlorophyll indexes. From each trifoliate leaf, we collected one side-leaf under normal conditions (well-watered, SMC of 30%). These leaves were quickly cut into two halves, one for CK measurement and the other for RNA purification, and both were frozen in liquid nitrogen and stored in −80^o^C until further use. Three leaves were also collected from the other two 3^rd^ trifoliate leaves of similar chlorophyll index to determine leaf relative water content under well-watered conditions (91±1%). The plants were then allowed to undergo drought treatment by withholding water until the SMC reached 5%. From the same 3^rd^ trifoliate leaves, from which one side-leaves had been collected as control well-watered samples, the other side-leaves were collected as drought-treated samples and prepared as described above for CK measurement and RNA purification. Three remaining leaves of the two trifoliates that were previously used for measuring relative water content under well-watered conditions were also collected separately to determine leaf relative water content under drought conditions (32±2%). All of the samples were collected at mid-day (11AM-1PM) and in three biological replicates.

### RNA isolation, DNAse treatment and cDNA synthesis for qRT-PCR

RNAs were purified using Trizol reagent (Invitrogen) according to a manufacturer-recommended protocol. DNAse I treatment and cDNA synthesis were performed as previously described [41].

### qRT-PCR and statistical analysis of the data

Primers for qRT-PCR were designed as previously described [41]. The *CYP2* gene was used as a reference gene in the expression profiling of soybean genes [42]. qRT-PCR reactions and data analyses were performed according to previously published methods [41]. Delta-CT method was used to calculate initial amount of target genes. When appropriate, a Student's *t*-test (one tail, unpaired, equal variance) was used to determine the statistical significance of the differential expression patterns between tissues and/or between treatments.

### Cytokinin analysis

Cytokinins were extracted and purified according to Dobrev and Kaminek [43] using reverse phase and ion exchange chromatography. Derivatives of *c*Z CKs were determined from retention time and the mass spectra of unlabeled standards and response ratio of their *t*Z counterparts. HPLC-MS analysis was performed as described by Dobrev *et al*.[44] using an HPLC (Ultimate 3000, Dionex) coupled to hybrid triple quadrupole /linear ion trap mass spectrometer (3200 Q TRAP, Applied Biosystems) set in selected reaction monitoring mode.

## Results and Discussion

### Identification and annotation of the *GmIPT* and *GmCKX* genes in soybean

We used a previously described genome-wide analysis method in soybean [37] to identify 17 putative *GmIPT* and 20 putative *GmCKX* genes which encode IPTs and CKXs proteins, respectively. Manual inspection led to the removal of several truncated sequences and we finally obtained a list of 14 and 17 putative soybean *GmIPT* and *GmCKX* genes, respectively. All of the GmIPT proteins were predicted to contain one or two IPP transferase domains (PF01715) and all of the GmCKX proteins contained the CK-binding (PF09265) and FAD-binding (PF01565) domains. Sequence features of the GmIPTs and GmCKXs are summarized in Tables 1 and 2.

**Figure 1:**
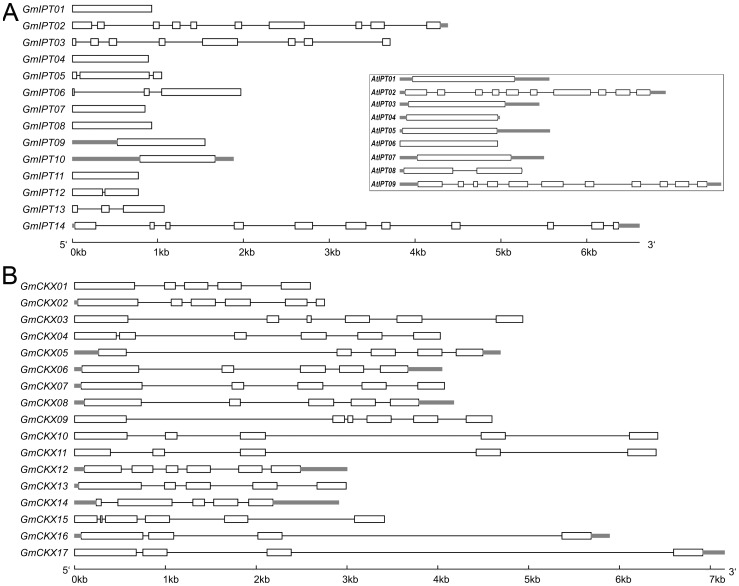
Organization of exons and introns of the soybean genes encoding IPTs and CKXs. (A) GmIPT proteins. (B) GmCKX proteins. Structure of the *Arabidopsis* genes encoding IPTs (box) is included in (A) for comparison.

Among the 14 *GmIPT*s, three members, namely *GmIPT02*, *GmIPT03* and *GmIPT14*, contain a large number of exons (10, 9 and 11, respectively), while five *GmIPT* members lack introns. The remaining six *GmIPT* genes possess two or three exons (Figure 1A, Table 1). This feature is also observed in genes encoding IPTs from *Arabidopsis* (Figure 1A, box). The group of *IPT* genes that lack introns or contain a small number of exons might have evolved from a prokaryotic ancestor and eventually some of the genes started gaining introns through the intronization process. Similar to their *AtIPT2* and *AtIPT9* counterparts in *Arabidopsis*, the *GmIPT* genes that contain high numbers of exons belong to the tRNA-dependent isopentenyltransferase group. Additionally, the rest of the GmIPTs were predicted to have at least one glycosylation site, except GmIPT01, 05 and 11. Each of the GmIPTs also possesses a signal peptide that directs it to various locations, such as mitochondria, chloroplast and other secretary pathways (Table 1).

**Table 1:** Soybean genes encoding putative IPT enzymes and their properties.

Gene name	Chromosome locus	Number of exons^a^	Domain feature (IPPT E-value)^b^		Family^c^	Length (aa)	Identity (%)^d^	Glycosylation sites^e^	TargetP^f^
*GmIPT01*	Glyma10g41990	−1	2.30E-19	-	AtIPT1/AtIPT8/AtIPT6	308	46.7/43.5/42.8	0	M/5
*GmIPT02*	Glyma11g19330	10	2.10E-44	-	AtIPT2	470	52	4	C/5
*GmIPT03*	Glyma12g09140	−9	4.90E-20	-	AtIPT2	321	36.2	2	− /5
*GmIPT04*	Glyma03g30850	−1	3.40E-21	4.80E-09	AtIPT3	296	48.5	2	C/4
*GmIPT05*	Glyma10g03060	−3	1.70E-20	5.40E-11	AtIPT3	315	46.7	0	−/4
*GmIPT06*	Glyma02g16750	3	9.90E-19	2.90E-11	AtIPT3	338	45.4	1	−/4
*GmIPT07*	Glyma19g33680	−1	5.20E-22	6.00E-10	AtIPT3	283	46.1	1	C/5
*GmIPT08*	Glyma17g02080	1	1.40E-15	3.20E-10	AtIPT5	311	51.1	2	−/3
*GmIPT09*	Glyma15g11040	−2	4.20E-18	1.00E-09	AtIPT5	342	48.1	4	S/5
*GmIPT10*	Glyma07g38620	−2	3.10E-17	1.80E-11	AtIPT5	292	48.2	3	−/2
*GmIPT11*	Glyma18g53460	1	3.70E-18	2.70E-10	AtIPT5/AtIPT7	256	36.2/34.5	0	−/3
*GmIPT12*	Glyma08g48020	−2	2.40E-18	-	AtIPT5/AtIPT7	246	32.3/31.5	2	−/2
*GmIPT13*	Glyma13g27990	3	6.50E-07	-	AtIPT5	211	29.5	1	−/3
*GmIPT14*	Glyma13g34680	11	2.90E-54	-	AtIPT9	448	58.9	1	M/4

a Minus signs represent genes located on opposite strand

b PFAM e-values for having the IPP transferase protein domain (PF01715) some GmIPTs contain more than one IPP domain

c Closest homologs from *Arabidopsis*

d Percentage of identical amino acids with the closest Arabidopsis homologs

e Glycosylation sites were predicted with NetNGly (http://www.cbs.dtu.dk/services/NetNGlyc/); 1-5, highest to lowest possibility

f Localization predicted with TargetP (http://www.cbs.dtu.dk/services/TargetP/); M, mitochondria; C, chloroplast; S, secretory pathways; “–”, not known location; 1–5, highest to lowest possibility

**Table 2:** Soybean genes encoding putative CKX enzymes and their properties.

Gene name	Chromosome locus	Number of exons^a^	FAD_binding_4^b^	CK-binding^b^	Family^c^	Length (aa)	Identity (%)^d^	Glycosylation sites^e^	TargetP^f^
*GmCKX01*	Glyma19g31620	5	1.50E-22	2.20E-112	AtCKX1/AtCKX6	544	59.1/60.8	4	C/5
*GmCKX02*	Glyma03g28910	7	1.20E-21	2.70E-69	AtCKX1/AtCKX6	551	49.2/49.7	4	C/5
*GmCKX03*	Glyma09g07190	−6	1.10E-21	4.00E-93	AtCKX2	533	45	6	M/5
*GmCKX04*	Glyma09g07360	6	1.40E-18	1.30E-118	AtCKX3	536	54.8	2	S/5
*GmCKX05*	Glyma13g16420	7	1.80E-12	1.70E-102	AtCKX3	429	42.1	7	−/2
*GmCKX06*	Glyma13g16430	−5	1.10E-23	1.00E-114	AtCKX3	535	54.3	1	S/3
*GmCKX07*	Glyma15g18560	5	2.60E-20	5.90E-118	AtCKX3	543	53.9	2	S/5
*GmCKX08*	Glyma17g06220	5	4.30E-23	1.60E-116	AtCKX3	535	55.8	2	S/1
*GmCKX09*	Glyma17g06230	−6	1.60E-20	4.90E-102	AtCKX3/AtCKX4	528	46.8/46.8	6	−/5
*GmCKX10*	Glyma06g03180	5	1.40E-22	9.40E-120	AtCKX5	518	64	1	S/2
*GmCKX11*	Glyma04g03130	5	3.20E-22	3.30E-120	AtCKX5	458	60.8	1	−/4
*GmCKX12*	Glyma09g35950	6	1.50E-20	1.10E-113	AtCKX6	534	68.6	3	S/1
*GmCKX13*	Glyma11g20860	5	5.20E-21	2.80E-114	AtCKX6	552	64.4	6	M/4
*GmCKX14*	Glyma12g01390	−6	2.20E-21	6.90E-67	AtCKX6	442	55.5	1	−/4
*GmCKX15*	Glyma04g05840	6	3.40E-11	1.90E-101	AtCKX7	494	58.6	2	−/2
*GmCKX16*	Glyma14g11280	−4	7.40E-29	1.20E-103	AtCKX7	513	61.5	3	−/2
*GmCKX17*	Glyma17g34330	4	3.10E-26	5.90E-103	AtCKX7	513	58.6	2	−/2

a Minus signs represent genes located on opposite strand

b PFAM e-values for having the indicated protein domains

c Closest homologs from *Arabidopsis*

d Percentage of identical amino acids with the closest Arabidopsis homologs

e Glycosylation sites were predicted with NetNGly (http://www.cbs.dtu.dk/services/NetNGlyc/); 1-5, highest to lowest possibility

f Localization predicted with TargetP (http://www.cbs.dtu.dk/services/TargetP/); M, mitochondria; C, chloroplast; S, secretory pathways; “–”, not known location; 1-5, highest to lowest possibility

The homology of soybean GmCKX proteins to their *Arabidopsis* counterparts was in the range from 45% to 68% (Table 2). The *GmCKX* genes contain 4 to 7 exons and encode proteins of 429 to 551 amino acid residues (Figure 1B, Table 2). Each of the GmCKXs contains an FAD-binding domain and a CK-binding domain with extremely high confidence (E-values < 3.4E-11 for FAD-binding domain and < 6.9E-67 for CK-binding domain). The GmCKX proteins were also predicted to harbor one to seven predicted sites for glycosylation, e.g. modifications which are responsible for the enhancement of enzyme activity [45], as well as a signal peptide that direct their transport.

### Phylogenetic analyses of GmIPT and GmCKX proteins

**Figure 2:**
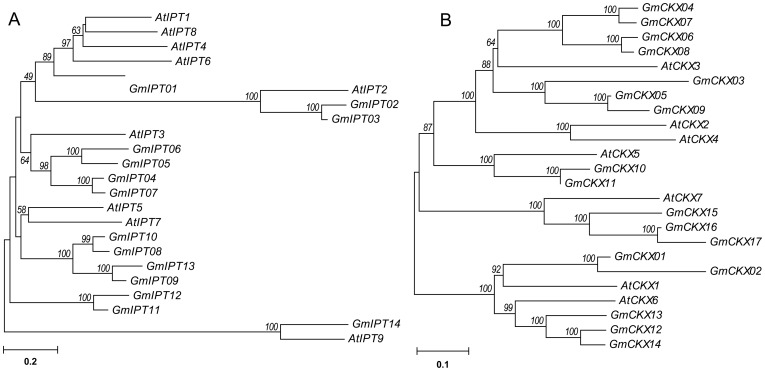
Evolutionary relationships of the soybean GmIPT and GmCKX proteins with their *Arabidopsis* counterparts. (A) GmIPT proteins. (B) GmCKX proteins. The bar indicates the relative divergence of the sequences examined. Bootstrap values higher than 50% are displayed next to the branch.

To uncover the evolutionary relationships among the soybean and *Arabidopsis* IPTs, as well as the soybean and *Arabidopsis* CKXs, we performed phylogenetic analyses using the neighbor-joining method implemented in MEGA software [39]. The protein sequences of the IPTs and CKXs from both species are highly similar (Figure 2). Three of the GmIPTs clustered with either AtIPT2 (GmIPT02 and GmIPT03) or AtIPT9 (GmIPT14), which are known to code for tRNA-dependent IPTs. As shown in Figure 2A, the tRNA-dependent IPTs and the ADP/ATP-dependent IPTs were highly diverse. It is also evident that the two species maintained different sets of *IPT* genes (Figure 2A). For example, the *AtIPT1*, *4*, *6*, *8* and *GmIPT01* genes appear to have originated from a common ancestor. After the speciation event, the genes expanded and were retained in *Arabidopsis* but not in soybean. In contrast, it is possible that the *AtIPT3* and *GmIPT04*, *05*, *06* and *07* genes might share a common ancestor but only the soybean genes expanded during evolution. A similar feature was also observed in genes coding for CKXs from *Arabidopsis* and soybean (Figure 2B). The GmCKX15, 16 and 17 were clustered with AtCKX07, which was recently reported to act on *c*Z-type CKs as a preferred substrate for degradation [46].

In addition, we observed that there are more CKX than IPT enzyme encoding genes (17 versus 14) in soybean, whereas the opposite was found in *Arabidopsis* (7 versus 9). This could be an advantage of evolution as more CKXs would enable soybean plants to robustly and precisely reduce CK content for a better response to adverse environmental conditions.

### Chromosomal distribution and duplications of *GmIPT* and *GmCKX* genes

**Figure 3:**
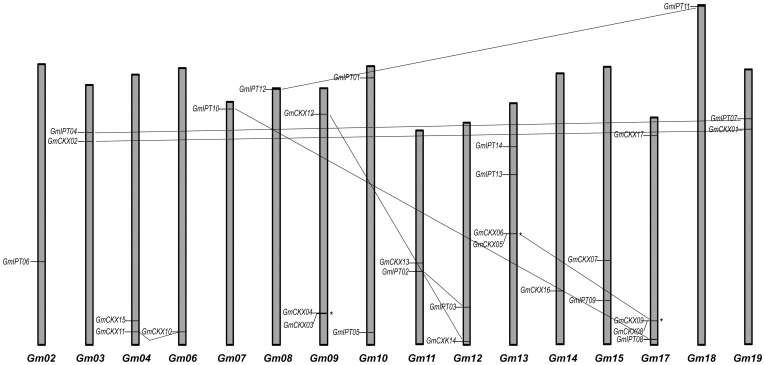
Graphical representation of chromosomal locations for putative *GmIPT* and *GmCKX* genes. Chromosomes 1, 5, 16 and 20 do not contain *GmIPT* and *GmCKX* genes. Tandemly duplicated genes are indicated by (*). Segmental duplicates are connected by inter-chromosomal lines.

Our analysis has indicated that soybean genes encoding GmIPTs and GmCKXs are distributed on various chromosomes, with exception of chromosomes I, V, XVI and XX (Figure 3). Gene duplication is also of interest because it is the source of genetic material for diversification [47]. Thus, we subsequently investigated the duplication patterns of *GmIPT* and *GmCKX* genes. We defined the genes as tandem duplicates if they were located within 20 loci from each other. For segmental duplicates, we analyzed the synteny blocks using an online tool (http://chibba.agtec.uga.edu/duplication/) [40]. We found three tandem duplicated pairs among the *GmCKXs* (Figure 3), namely *GmCKX03* and *GmCKX04* on chromosome IX; *GmCKX05* and *GmCKX06* on chromosome XIII; and *GmCKX08* and *GmCKX09* on chromosome XVII. No tandem duplication was found among the *GmIPTs*. Segmental duplicates were found in both *GmIPT* and *GmCKX* gene families. As evidenced by synteny analysis, four pairs of *GmIPT*s were formed by segmental duplication: *GmIPT04* (Chr. III) and *GmIPT07* (Chr. XIX); *GmIPT10* (Chr. VII) and *GmIPT08* (Chr. XVII); *GmIPT12* (Chr. VIII) and *GmIPT11* (Chr. XVIII); and *GmIPT13* (Chr. XIII) and *GmIPT09* (Chr. XV) (Figure S3A). Among 17 *GmCKX* genes, five pairs were formed by segmental duplication: *GmCKX02* (Chr. III) and *GmCKX01* (Chr. XIX); *GmCKX11* (Chr. IV) and *GmCKX10* (Chr. VI); *GmCKX12* (Chr. IX) and *GmCKX14* (Chr. XII); *GmCKX05* (Chr. XIII) and *GmCKX06* (Chr. XIII); and *GmCKX08* (Chr. XVII) and *GmCKX09* (Chr. XVII), the last two pairs being first formed by tandem duplication and then by segmental duplication (Figure 3; Figure S3B). The differences in duplication patterns of the two gene families may reflex their contrasting functions in regulating CK levels in soybean plant.

### Stress-inducible *cis*-regulatory elements in the promoter regions of *GmIPT* and *GmCKX* genes

*Cis*-regulatory elements, which are located in the upstream regions of genes and act as the binding sites for TFs, have essential roles in determining the tissue-specific or stress-responsive expression patterns of genes. Over the years, extensive promoter analyses have identified a number of stress-responsive *cis*-elements, which are important molecular switches involved in the transcriptional regulation of a dynamic network of gene activities controlling abiotic stress responses [4], [48], [49]. Increasing evidence has demonstrated a positive correlation between multi-stimulus responsive genes and *cis*-element density in upstream regions [50]-[52]. Therefore, in order to identify possible candidates among *GmIPT* and *GmCKX* genes which are involved in abiotic stress responses in soybean plants, we performed a search for the existence of the eleven known stress-responsive *cis* element(s) in the 1000-bp promoter region upstream of the transcription start site of each GmIPT and GmCKX encoding gene. As shown in Table S1, three of the known abiotic-stress responsive *cis-*elements were found in the promoters of *GmIPT* gene family members. Specifically, MYCR (MYC recognition site) was found in five *IPT* members (*GmIPT04, 05, 08, 11* and *12*), ZFHDR (zinc finger homeodomain recognition site) was found in *GmIPT01* and ICEr2 (Inducer of CBF expression 2 recognition site) was found in *GmIPT07*. Among the promoters of the *GmCKX* family, we detected five abiotic-stress responsive *cis-*elements, which are ABRE (ABA-responsive element), MYBR (MYB recognition site), MYCR, ZFHDR and ICEr2 (Table S1). Although distributed on promoters of both *GmIPT*s and *GmCKX*s, on the average there were 0.4 and 1.2 abiotic stress-inducible *cis*-elements per promoter of genes encoding GmIPT and GmCKX, respectively. This evidence suggested that upregulation of *GmCKX*s, thereby reduction of CK levels, could be the major event in soybean plants under abiotic stresses. In agreement with our data, in *Arabidopsis* three out of seven *AtCKX* genes were found to be upregulated by salt stress [11]. The upregulated *CKX* genes, either in *Arabidopsis* or soybean, may contribute to faster degradation of the accumulated CKs upon exposure to environmental stresses, thereby enhancing plant adaptation to adverse stress conditions.

### Expression of *GmIPT* and *GmCKX* genes in vegetative organs at early and late developmental stages under normal growth conditions

Tissue-specific and development stage-related expression data are useful in the identification of genes that are involved in defining the precise nature of individual tissues in a given developmental stage. Moreover, the mechanisms controlling the response to drought stress may be associated with root- and/or shoot-related traits. For instance, suppression of shoot growth and/or promotion of primary root growth are considered morphological adjustments enabling plants to adapt better to drought stress [1], [3], [11], [12]. CKs are well-known to positively regulate shoot growth but negatively regulate root growth. As a result, the appropriate control of shoot- and root-related morphological traits, via the modulation of endogenous CK levels prior to the occurrence of a stress, as a preventive measure, is a promising approach for developing economically important drought-tolerant crops [12], [32]. Apart from their biochemical characteristics, tissue-specific and development stage-related expression of the CK metabolic genes indicate their functional specification and potential utility for the genetic engineering of specific traits. A well-known example is that among the seven *Arabidopsis* ATP/ADP *IPT* genes, *IPT1*, *3*, *5* and *7* are expressed in the vegetative phase and *IPT4*, *6* and *8* are not. Thus, the *ipt1,3,5,7* quadruple mutant has reduced active CK levels which results in morphological adjustment (shorter shoot and longer primary root), hypersensitivity to ABA and enhanced cell membrane integrity contributing to enhanced drought-tolerant phenotypes [11], [19].

**Figure 4:**
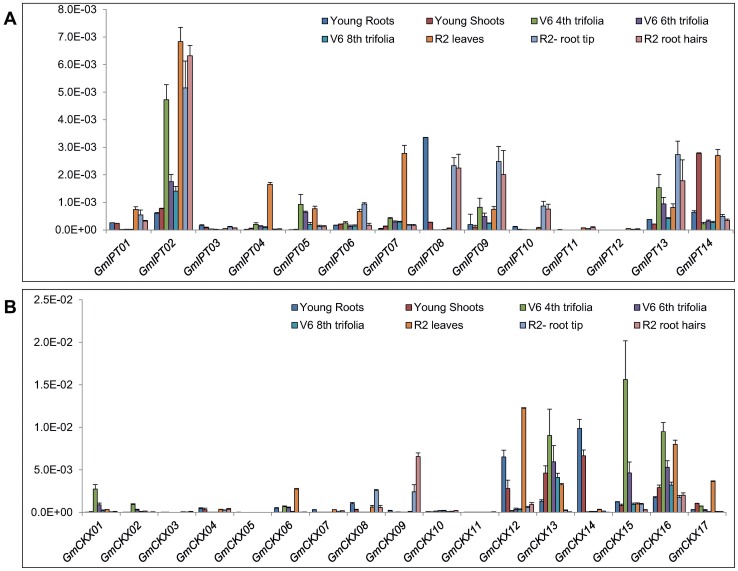
Expression of *GmIPT* and *GmCKX* genes in vegetative tissues of soybean plants at various development stages at normal conditions. (A) *GmIPT* genes. (B) *GmCKX* genes.

Thus, in order to obtain the first glance on the roles of each of the *GmIPT* and *GmCKX* genes during vegetative development, we designed primers (Table S2) and quantified the transcript levels of these genes by qRT-PCR in the roots of 12-d-old young seedlings and R2 soybean plants, as well as in the shoots of young seedlings, leaves of V6 and R2 soybean plants (Figure 4). As a result of the expression analyses, we found that not all of the *GmIPT* and *GmCKX* genes were expressed in each of the organs; a phenomenon which was also observed in *Arabidopsis* [53]. For example, tRNA-type *GmIPT02* was highly expressed among all tissues examined, meanwhile *GmIPT08* was only highly expressed in root tissues and *GmIPT04* and *07* were highly expressed only in reproductive leaves (Figure 4A). Expression of three other *GmIPT* genes (*GmIPT03*, *11* and *12*) were barely detected. Among the ATP/ADP-type GmIPT encoding genes, *GmIPT05*, *07*, *09* and 134xhibited gradual increases in transcript abundance in the leaves of V6 stage plants in correlation with the age of the trifoliate leaves (Figure 4A). In regard to the expression in root tissues, *GmIPT08* was found to be the major transcript in the roots of young seedlings while *GmIPT02* mRNA had the highest abundance in the roots of R2 plants (Figure 4B). Taken together, these data indicated that soybean requires different *IPT* genes for the biosynthesis of CKs in different organs and at different developmental stages.

CKXs are the key enzymes involved in the regulation of CK levels in plants for the maintenance or reestablishment of CK homeostasis. In order to determine which *GmCKX* gene(s) may play important regulatory roles in specific organ(s) of the soybean plants, we measured the *GmCKX* transcripts in various tissues/organs. Among 17 *GmCKX* genes, *GmCKX13*, *15* and *16* were highly expressed in all tissues examined, while the expression of four other *GmCKX*s, *GmCKX03*, *05*, *10* and *11*, was hardly detected. At the same time, *GmCKX14* transcript was only found in young seedling tissues (Figure 4B). In soy leaves, *GmCKX12*, *13*, *14* and *16* were detected as major transcripts in young seedling shoots, *GmCKX13*, *14*, *15* and *16* mRNAs were the most abundant in the V6 leaves and *GmCKX12* and *16* expression levels were the highest in R2 leaves (Figure 4B). As for root tissues, *GmCKX12* and *14* were the major transcripts in the young seedling roots, while *GmCKX09* and *16* were most abundant in the R2 auxilary roots and root hairs. On the other hand, *GmCKX15* was the major transcript in R2 auxiliary roots only (Figure 4B). Additionally, the major transcripts of *GmIPT* and *GmCKX* genes determined in our study were also highly expressed in various tissues as reported by Libault and co-workers [54] using Illumina transcriptome sequencing (Figure S4), suggesting a good correlation between the qRT-PCR and Illumina transcriptome sequencing methods in expression profiling.

### Expression of *GmIPT* and *GmCKX* genes in vegetative organs at early and late developmental stages under dehydration/drought stress

**Figure 5:**
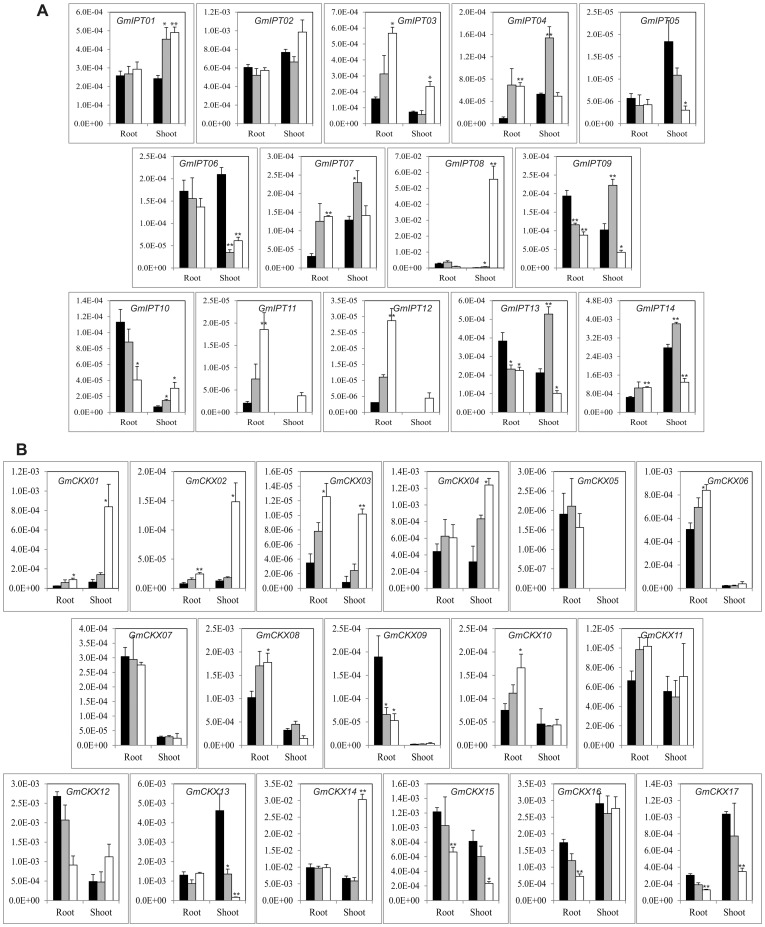
Expression profiles of *GmIPT*s and *GmCKX*s in the roots and shoots of 12-day-old soybean seedlings under normal and dehydration conditions. (A) *GmIPT* genes. (B) *GmCKX* genes. Black bars; expression under normal condition (0 h); gray bars, expression under 2 h dehydration; white bars, expression under 10 h dehydration.

**Figure 6:**
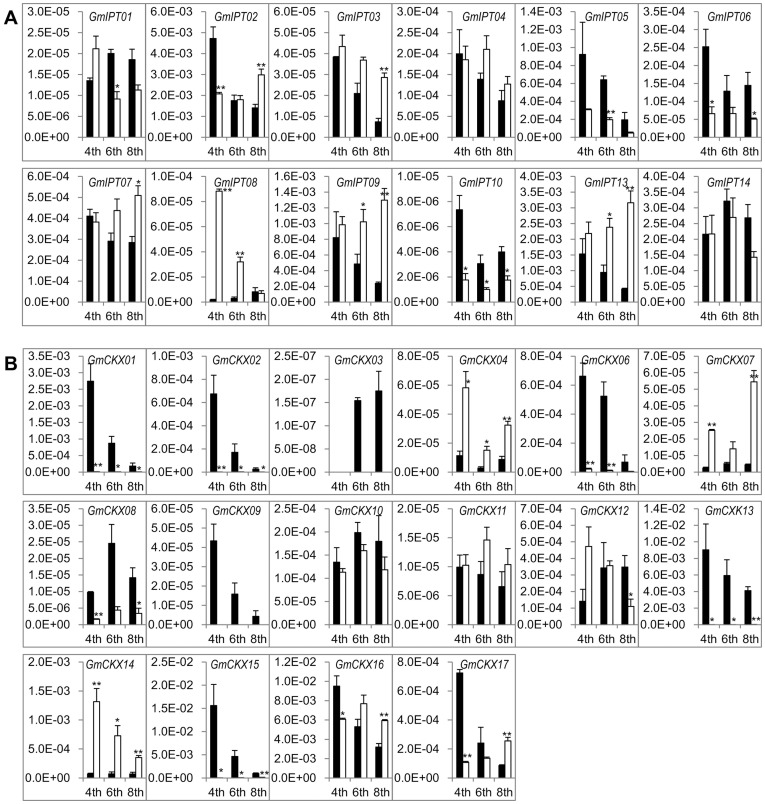
Expression profiles of *GmIPT*s and *GmCKX*s in V6 trifoliate leaves (4^th^, 6^th^ and 8^th^) under normal and drought conditions. (A) *GmIPT* genes. (B) *GmCKX* genes. Black bars; expression under normal condition; white bars, expression under drought condition.

**Figure 7:**
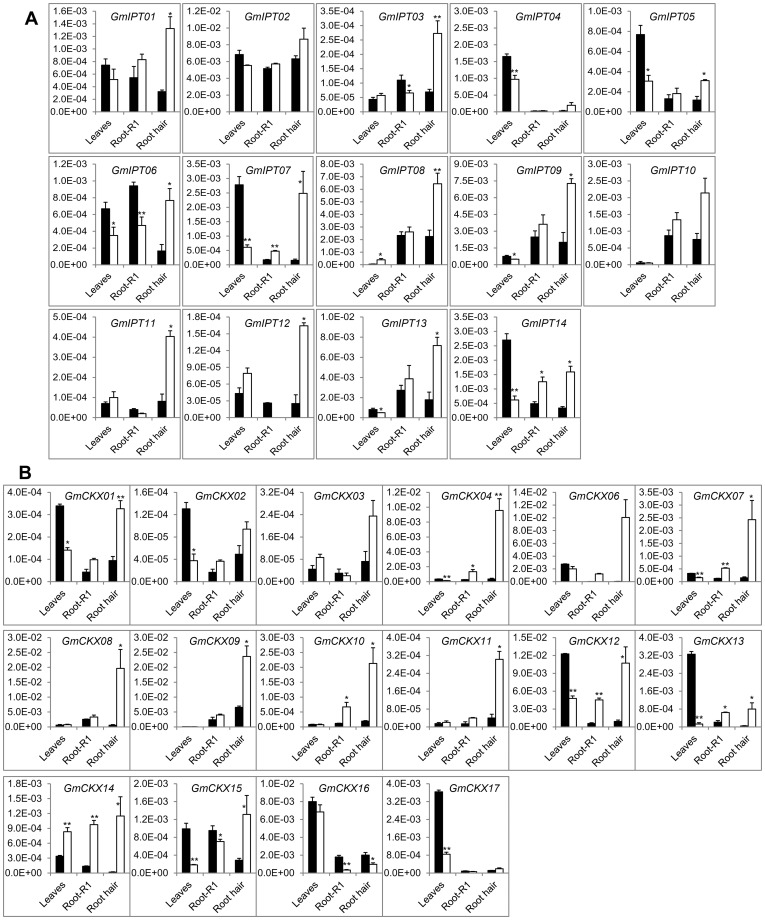
Expression profiles of *GmIPT*s and *GmCKX*s in the leaves, roots and root hairs of soybean plants at reproductive stage under normal and drought conditions. (A) *GmIPT* genes. (B) *GmCKX* genes. Black bars; expression under normal condition; white bars, expression under drought condition.

Among the *IPT* transcripts expressed in remarkably high abundance, the *GmIPT08* transcript was consistently increased in the leaves and young seedling shoots under drought or dehydration conditions (Figures 5A, 6A and 7A). The induction level of this transcript was the highest in young seedling shoots (∼300-fold, Figure 5A). In the V6 leaves, the induction levels were correlated with the age of the trifoliate leaves; the older the leaf is the higher the induction (Figure 6A). In R2 leaves, *GmIPT08* was the only gene whose expression was significantly induced (∼7-fold) by drought (Figure 7A). Other *GmIPT* genes, which were significantly induced by drought in the V6-stage leaves, are *GmIPT09* and *13*, and the degree of induction of these genes was higher in the younger trifoliate leaves (Figure 6A). As for the drought-repressible *IPT* genes identified in the leaf tissues, the *GmIPT05* transcripts were repressed by drought in the leaves of various stages (Figures 5A, 6A and 7A). In the R2-stage leaves, expression of almost all highly expressed *GmIPT* genes, except that of *GmIPT08*, was repressed by drought. The most substantial repression was observed in case of the *GmIPT07* gene (Figure 7A). In the roots of young seedlings, the most abundant *GmIPT08* transcript (Figure 4A) was slightly induced by dehydration after 2 h of treatment, being subsequently repressed after 10 h of dehydration (Figure 5A). In the R2 auxiliary roots, the transcripts with high abundance did not change significantly upon dehydration. These data are in contrast with the observation in root hairs where all major transcripts, such as *GmIPT01*, *07*, *08*, *09* and *13*, were induced upon 5-h dehydration (Figure 7A).

As for the *GmCKX* genes, five *GmCKX*s (*GmCKX01*, *02*, *06*, *13* and *15*) were found to be severely reduced at transcriptional level by drought in the V6 leaves (Figure 6B), and four of which, except *GmCKX06*, were also significantly downregulated in R2 leaves under drought (Figure 7B). Nevertheless, in young seedling shoots, the expression of *GmCKX01* and *02* was induced by dehydration (Figure 5B). In addition, the *GmCKX14* transcript was found to be significantly induced by drought in various tissues at different stages. Furthermore, most of the *GmCKX*s were induced by drought in the R2 roots (Figure 7B). Among 17 *GmCKX* genes, only *GmCKX16*, whose encoded protein might be involved in degradation of *c*Z-type CKs as its AtCKX7 ortholog [46], was significantly upregulated by drought in the roots of the R2 stage soybean plants (Figure 7B).

### Expression of *GmIPT* and *GmCKX* genes in reproductive tissues under normal growth conditions

**Figure 8:**
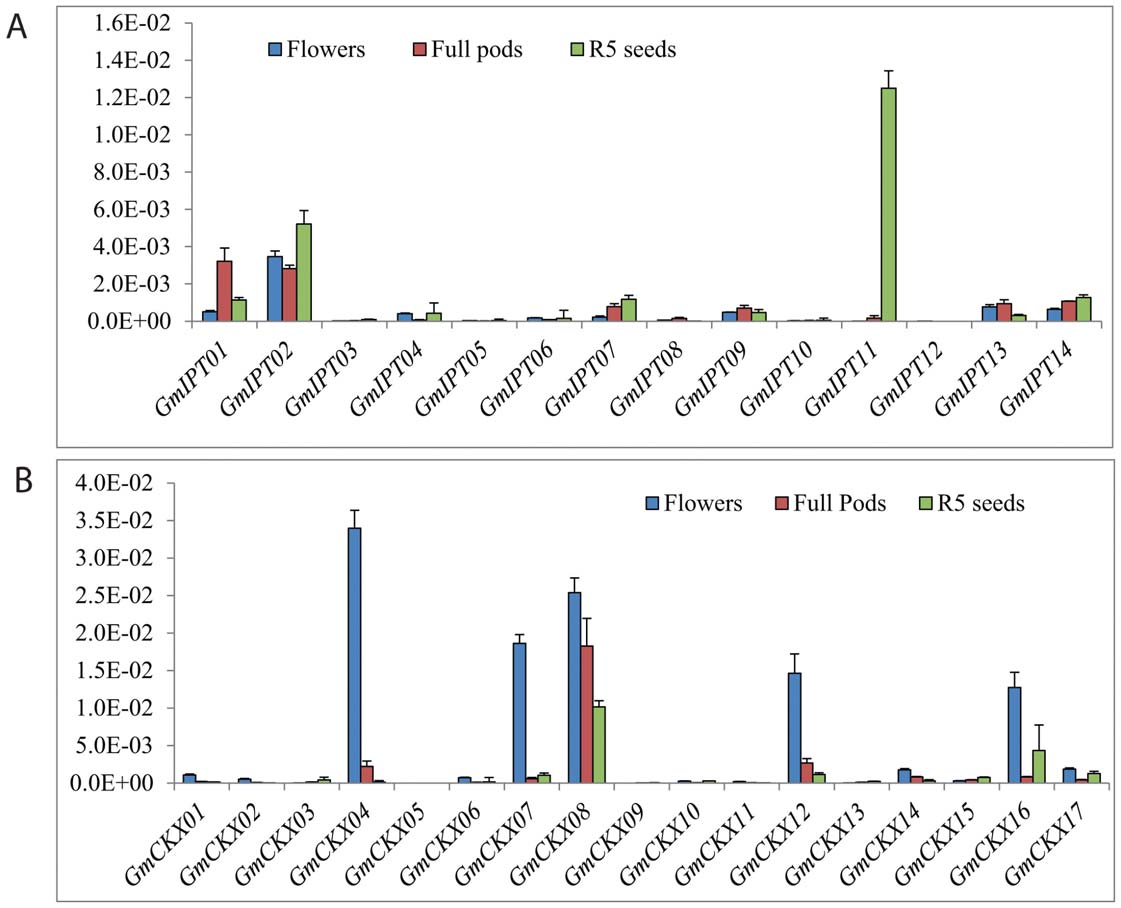
Expression profiles of *GmIPT*s and *GmCKX*s in flowers (R1-R2), full pods (R4) and seeds (R5) of soybean plants grown under normal conditions. (A) *GmIPT* genes. (B) *GmCKX* genes.

Strong lines of evidence have also suggested that CKs play an important role in the development of reproductive organs and seed yield. Disruption of *CKX3* and *CKX5* genes in *Arabidopsis* resulted in higher CK levels, which subsequently led to larger inflorescences and floral meristems, increased size of the WUSCHEL expression domain, supernumerary ovules and increased seed yield of the *ckx3,5* double mutant plants [55]. An increase in CK accumulation caused by a null mutation in the *OsCKX2* gene was also shown to enhance the size of inflorescence meristems and increase the number of reproductive organs, resulting in enhanced grain yield [56]. Therefore, to gain an insight into the CK metabolism in reproductive organs, we analyzed the expression of CK metabolic genes in flowers, full pods and R5 seeds. Results shown in Figure 8A indicated that *GmIPT02* is ubiquitously expressed in all three reproductive tissues examined while five other *GmIPT*s (*GmIPT03*, *05*, *08*, *10* and *12*) were not expressed. *GmIPT02* was the major transcript in flowers, *GmIPT01* and *02* expressed in the greatest abundance in full pods while *GmIPT01*, *02* and *11* were the most abundant transcripts in R5 seeds. The variation in the *GmIPT* transcript levels in flowers, pods and R5 seeds suggested that each of these organs required different *GmIPT* genes for CK biosynthesis. *GmIPT02* and *GmIPT11* might play the most important role in flowers and R5 seeds, respectively, while *GmIPT01* and *02* appear to be equally important in full pods as judged by their abundant expression.

The expression levels of *GmCKX* genes were also determined in flowers, pods and R5 seeds (Figure 8B). In flowers, five out of 17 *GmCKX* mRNAs were dominant, including *GmCKX04*, *07*, *08*, *12* and *16*. Unlike in flowers, *GmCKX08* transcript was in the highest abundance measured in pods. In R5 seeds, *GmCKX08* was still the most highly abundant transcript, while the second most abundant one was *GmCKX16*. It is noteworthy to mention that the expression of nine out of 17 *GmCKX*s was hardly detected in the three reproductive tissues examined (Figure 8B), including *GmCKX13*, which were highly expressed in various vegetative tissues (Figure 4B). Our data suggested that *GmCKX08* is perhaps the major regulator of CK levels in reproductive organs. On the other hand, in flower tissues, the concerted action of at least five GmCKXs is required for maintaining CK homeostasis.

In addition, our expression data presented in Figures 4, 5, 6, 7, and 8 also indicated that a few duplicated gene pairs have undergone expression divergence (*GmIPT08* and *GmIPT10*; *GmCKX05* and *GmCKX06*; and *GmCKX12* and *GmCKX14*), whereas the majority of the duplicated pairs have not changed their expression patterns (*GmIPT04* and *GmIPT07*; *GmIPT11* and *GmIPT12*; *GmIPT09* and *GmIPT13*, *GmCKX01* and *GmCKX02*; *GmCKX08* and *GmCKX09*; and *GmCKX10* and *GmCKX11*). The data also showed that although the majority of *GmIPT* and *GmCKX* genes exhibited similar stress-responsiveness to that of their respective *Arabidopsis* orthologous genes, several *GmIPT* and *GmCKX* genes have undergone transcriptional divergence during evolution (Table S3).

### CK metabolites in various organs of soybean plants under normal and drought conditions

**Figure 9:**
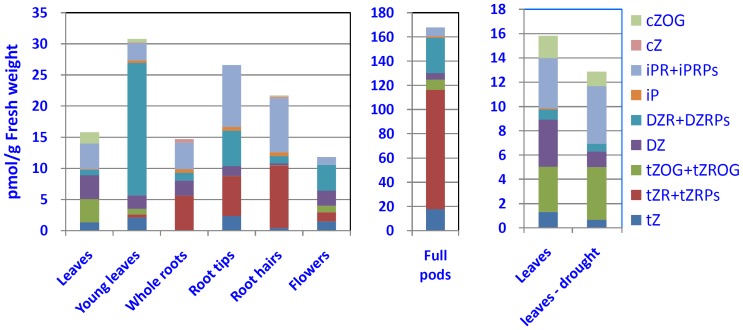
CK content in various tissues of soybean plants under well-watered and drought stress conditions. Leaves, fully developed R2 leaves of well-watered soybeans (RWC of 91±1%); Young leaves, not-fully expanded R2 leaves of well-watered soybeans; Whole roots, hydroponic parts of root segments, including auxiliary roots, lateral roots and root hairs, of R2 soybean plants grown in a semi-hydroponic manner (Figure S2); Root tips, hydroponic parts of root tips of R2 soybean plants grown in a semi-hydroponic manner (Figure S2); Root hairs, hydroponic parts of root hairs of R2 soybean plants grown in a semi-hydroponic manner (Figure S2); Flowers, flowers of well-watered R1-R2 soybeans; Full pods, R4 full pods of well-watered soybeans; Leaves-drought, fully developed R2 leaves of drought-stressed soybeans (RWC of 32±2%).

To gain an overall image of CK functions in the development of soybean plants, we quantified the CK metabolites in various tissues/organs collected from soybean plants at various stages as described in Materials and Methods (Table S4). The most remarkable result is that compared with the levels of *t*Z-type and iP-type CKs, the levels of *c*Z-type CKs were significantly lower in almost all of the tissues/organs examined (Figure 9; Table S4), despite the fact that tRNA-type GmIPT encoding genes were found to be highly expressed (Figure 4). These data are similar to the situation observed in *Arabidopsis* [11], [19]. Our results added up to a recent study by Gajdosova *et al.* [46] which indicated that *c*Z-type CKs occur ubiquitously across plant kingdom and their abundance is, perhaps, correlated with life strategy rather than with evolutionary complexity. In the vegetative organs, such as leaves, the total content of CKs, as well as the CK compositions, also varied with age. For example, the total CK content in young R2 leaves was higher than that in fully developed R2 leaves. This increase was mainly result of an increase in the DZR and DZRP (Figure 9, left panel), suggesting that the DZ-type CKs are also significantly produced in young leaves of soybean, in addition to dormant seeds and apical buds as observed in bean [57], [58]. Increasing evidence suggests that several CK receptor kinases, such as the AHK3 of *Arabidopsis* or the ZmHK2 of maize, have affinity to DZ-type CKs,[59]-[61] suggesting that the DZ-type CKs might have biological functions in plants. Previously, we showed that in soybean there are two GmHKs, the GmHK12 and 13, which have high homology to the AHK3 [37]. Taken together, the DZ-type CKs might be biologically active in soybean and their variation in levels may reflex their active roles in regulating plant growth and development of soybean plants.

In the R2 plants, the levels of *t*Z and *t*ZR(P) were higher in young leaves than in the more mature fully expanded leaves. The mature leaves accumulated more *t*Z deactivation products (*t*Z(R) *O*-glucosides). It appears that CK metabolites are organ-specific. For example, *t*Z, its riboside and their immediate precursors (*t*ZRPs) are abundant in roots, especially in root tips and hairs, which are the primary sites of CK biosynthesis. *t*Z, the most physiologically active CK in the stimulation of cell division, is also relatively abundant in other rapidly growing tissues (e.g. young leaves, flowers and pods). In addition, flowers and pods also contain very high levels of *t*ZR and *t*ZRPs as well (Figure 9, left and middle panels). On the contrary, *t*ZRPs were not detected in fully developed leaves (Figure 9, left panel) at reproductive stage. Physiologically inactive *t*ZOG and *t*ZROG were more abundant in mature leaves and were less abundant in young leaves, flowers and pods. These data suggest that a large amount of *t*Z-type CKs are in storage forms in these organs. CK *O*-glucosides were undetectable in roots. Pods exhibited the highest level of total CKs, 5- to 10-fold higher than that found in other organs. These data suggest a complex function, in which different compounds account for development of different organs at different developmental stages.

Since an increasing amount of evidence suggests that CKs play an important role in the regulation of the drought response, we examined the effects of drought stress on overall CK metabolism in soybean plants by comparing the CK content in R2 leaves under control and drought conditions. A CK analysis was performed in leaves collected at the R2 stage, because this stage is considered critical, where drought stress may cause detrimental effects on soybean productivity (http://www.uwex.edu/ces/ag/issues/drought2003/soybeansrespondstress.html). The results shown in Figure 9 (right panel) indicated that CK content was lower in drought stressed leaves, which is in agreement with the results observed in other plant species [25], [62]. The most pronounced changes were observed with active CKs, *t*Z and DZ, which were significantly reduced in the R2 leaves upon drought treatment. The total content of *t*Z-type CKs was relatively unchanged. Our data suggest that a decrease in DZ content, which is associated with the downregulation of the majority of the *GmIPT* genes and the upregulation of *GmCKX03* and *GmCKX14* (Figure 7), contributes significantly to the overall reduction of CK content in drought stressed soybean leaves. Additionally, DZ may be involved in regulation of drought stress responses through GmHK12, which has high homology to AHK3. Both *GmHK12* and *AHK3* were upregulated by drought stress, and AHK3 was shown to act as a negative regulator of drought stress signaling in *Arabidopsis* [5], [36], [37].

### Conclusions

Research in the last several years has indicated that CKs play an essential role in the regulation of plant adaptation to various environmental stresses, including drought [63]. Repression of CK metabolism under adverse stress conditions, which leads to a downregulation of CK signaling, is known as one of the mechanisms used by plants to adapt to adverse environmental conditions [32]. The results of this study provided the first insight into the previously uncharacterized CK metabolic genes encoding GmIPTs, which are involved in the rate-limiting step of CK biosynthesis. In addition, we also investigated *GmCKX*s, encoding the main CK degrading enzymes, which contribute to maintenance or reestablishment of CK homeostasis. Throughout our investigation, we placed a particular emphasis on their tissue-specific and/or drought-responsive expression. Collectively, these data enable us to understand the molecular mechanisms regulating CK homeostasis in various tissues/organs at different developmental stages under both normal and drought stress conditions.

In addition, appropriate modulations of CK levels, based upon the knowledge of mechanisms regulating CK metabolism and CK homeostasis, represent promising approaches for the genetic engineering of drought-tolerant economically important crops [32], [63]. A reduction in CK content in roots by the constitutive overexpression of a *CKX* gene in a root-specific manner can improve drought tolerance by enhancing root biomass [12]. On the other hand, an increase in CK content just prior to the onset of senescence was also shown to improve leaf longevity and photosynthetic capacity under drought stress, thereby enhancing drought tolerance without yield penalties [33], [64]. Therefore, our study has generated a solid foundation for the identification of candidate genes for future studies which aim to manipulate CK metabolism to appropriate levels and ultimately contribute to the development of improved drought-tolerant transgenic soybeans.

## Supporting Information

Figure S1 **Drought treatment of soybean plants grown in pots at the V6 stage.** (A) Three soybean plants were grown in each pot to V6 stage (four weeks). The V6 plants (containing 7 trifoliate leaves, unifoliate leaves still remained) were withheld from watering; during this time, volumetric soil moisture content (SMC) and room relative humidity were recorded. (B) At the 6^th^ day after withholding water, the leaves were collected from both well-watered and drought-stressed plants. Trifoliate leaves 3^rd^, 5^th^ and 7^th^ were used for measuring leaf relative water content, while trifoliate leaves 4^th^, 6^th^ and 8^th^ were used for RNA extraction. After the leaves were collected, the drought-stressed plants were re-watered and monitored to ensure that all drought-treated plants survived after drought treatment. Figure S1C shows well-watered and drought-stressed soybean plants just prior to collecting the leaves.
(DOC)

Figure S2 **Growth of soybean plants under semi-hydroponic conditions.** Soybean plants were allowed to grow under semi-hydroponic conditions for the collection of root tissues. Detached roots were used for dehydration treatment.
(DOC)

Figure S3 **Synteny analysis of** ***GmIPT*** **and** ***GmCKX*** **genes.** (A) Synteny analysis revealed evidence of the segmental duplication among several *GmIPT* genes in soybean. (B) Synteny analysis revealed evidence of the segmental duplication among several *GmCKX* genes in soybean.
(DOC)

Figure S4 **Clustering analysis of tissue-specific expression profiles of** ***GmIPT*** **and** ***GmCKX*** **genes.** (A) Expression data (normalized Illumina-Solexa read numbers) collected from Libault et al. (2010) [54]. (B) Expression data from our study. Both two data sets showed that *GmIPT02* was highly expressed among the tissues examined and that *GmCKX04*, *07*, *08*, *12* and *16* were highly expressed in flowers, suggesting a good agreement between our qRT-PCR data and the data derived from Illumina-Solexa cDNA-sequencing study.
(DOC)

Table S1 **Number of abiotic-stress inducible *cis*-elements in the promoters of *GmIPT*s and *GmCKX*s.**
(DOC)

Table S2 **Primers used for qRT-PCR.**
(DOC)

Table S3 **Drought/dehydration-responsiveness of the soybean and *Arabidopsis IPT* and *CKX* genes.**
(DOC)

Table S4 **CK contents in various soybean tissues under normal and drought stress conditions.** (A) Concentration of individual CK metabolites in various soybean tissues. (B) CK contents in various soybean tissues in group of compounds.
(DOC)
