# Supplementary material for: Identification and Expression Analysis of Cytokinin Metabolic Genes in Soybean under Normal and Drought Conditions in Relation to Cytokinin Levels
Source: PLoS One. 2012 Aug 10;7(8):e42411. doi: 10.1371/journal.pone.0042411 (PMC3416864; doi:10.1371/journal.pone.0042411)
Supplement: Figure S1 — Drought treatment of soybean plants grown in pots at the V6 stage. (A) Three soybean plants were grown in each pot to V6 stage (four weeks). The V6 plants (containing 7 trifoliate leaves, unifoliate leaves still remained) were withheld from watering; during this time, volumetric soil moisture content (SMC) and room relative humidity were recorded. (B) At the 6th day after withholding water, the leaves were collected from both well-watered and drought-stressed plants. Trifoliate leaves 3rd, 5th and 7th were used for measuring leaf relative water content, while trifoliate leaves 4th, 6th and 8th were used for RNA extraction. After the leaves were collected, the drought-stressed plants were re-watered and monitored to ensure that all drought-treated plants survived after drought treatment. Figure S1C shows well-watered and drought-stressed soybean plants just prior to collecting the leaves. (DOC) [file pone.0042411.s001.doc]

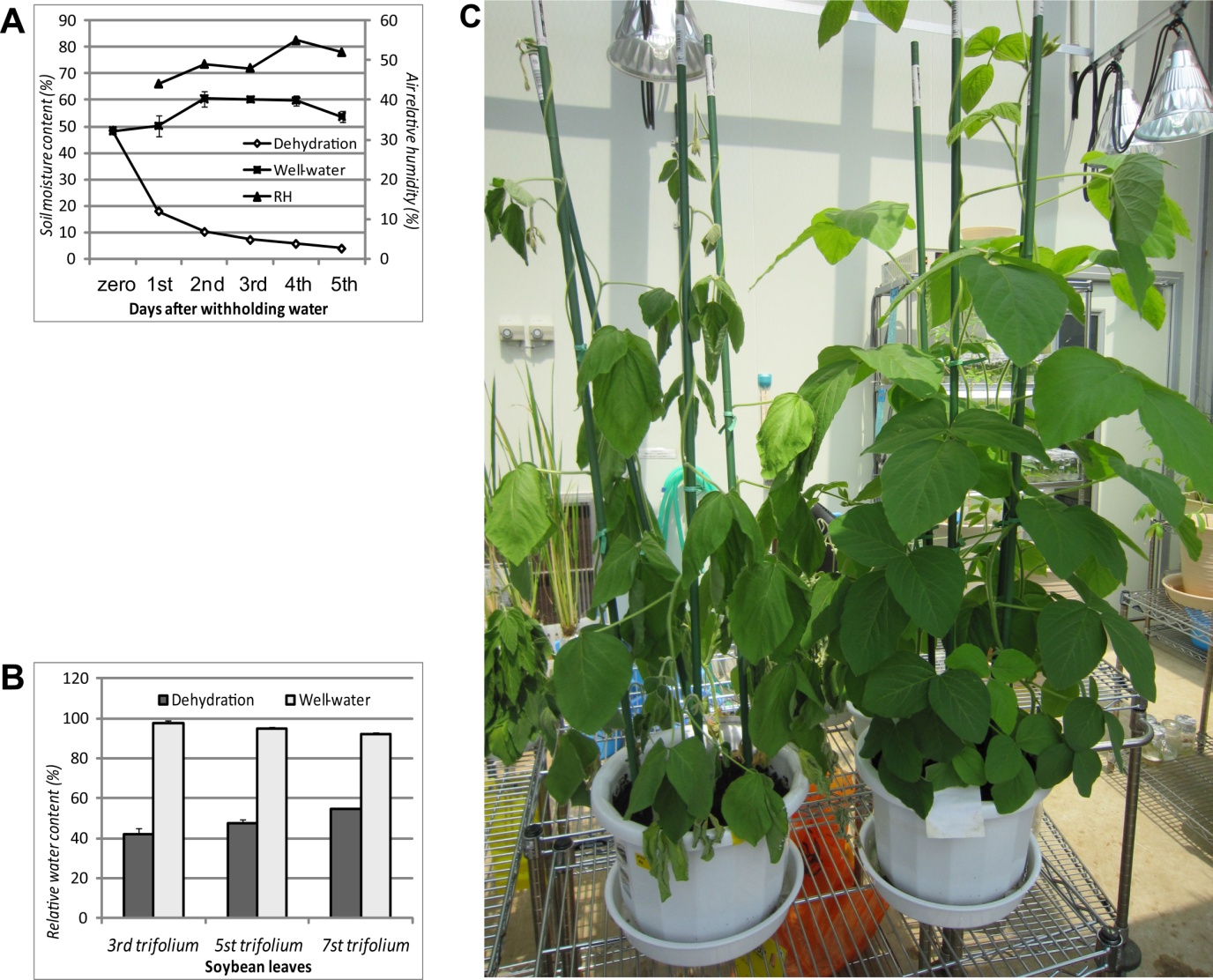


**Figure S1.** Drought treatment of soybean plants grown in pots at the V6 stage. (A) Three soybean plants were grown in each pot to V6 stage (four weeks). The V6 plants (containing 7 trifoliate leaves, unifoliate leaves still remained) were withheld from watering; during this time, volumetric soil moisture content (SMC) and room relative humidity were recorded. (B) At the 6^th^ day after withholding water, the leaves were collected from both well-watered and drought-stressed plants. Trifoliate leaves 3^rd^, 5^th^ and 7^th^ were used for measuring leaf relative water content, while trifoliate leaves 4^th^, 6^th^ and 8^th^ were used for RNA extraction. After the leaves were collected, the drought-stressed plants were re-watered and monitored to ensure that all drought-treated plants survived after drought treatment. (C) Well-watered and drought-stressed soybean plants just prior to collecting the leaves.
