# Supplementary material for: Identification and Expression Analysis of Cytokinin Metabolic Genes in Soybean under Normal and Drought Conditions in Relation to Cytokinin Levels
Source: PLoS One. 2012 Aug 10;7(8):e42411. doi: 10.1371/journal.pone.0042411 (PMC3416864; doi:10.1371/journal.pone.0042411)
Supplement: Figure S2 — Growth of soybean plants under semi-hydroponic conditions. Soybean plants were allowed to grow under semi-hydroponic conditions for the collection of root tissues. Detached roots were used for dehydration treatment. (DOC) [file pone.0042411.s002.doc]

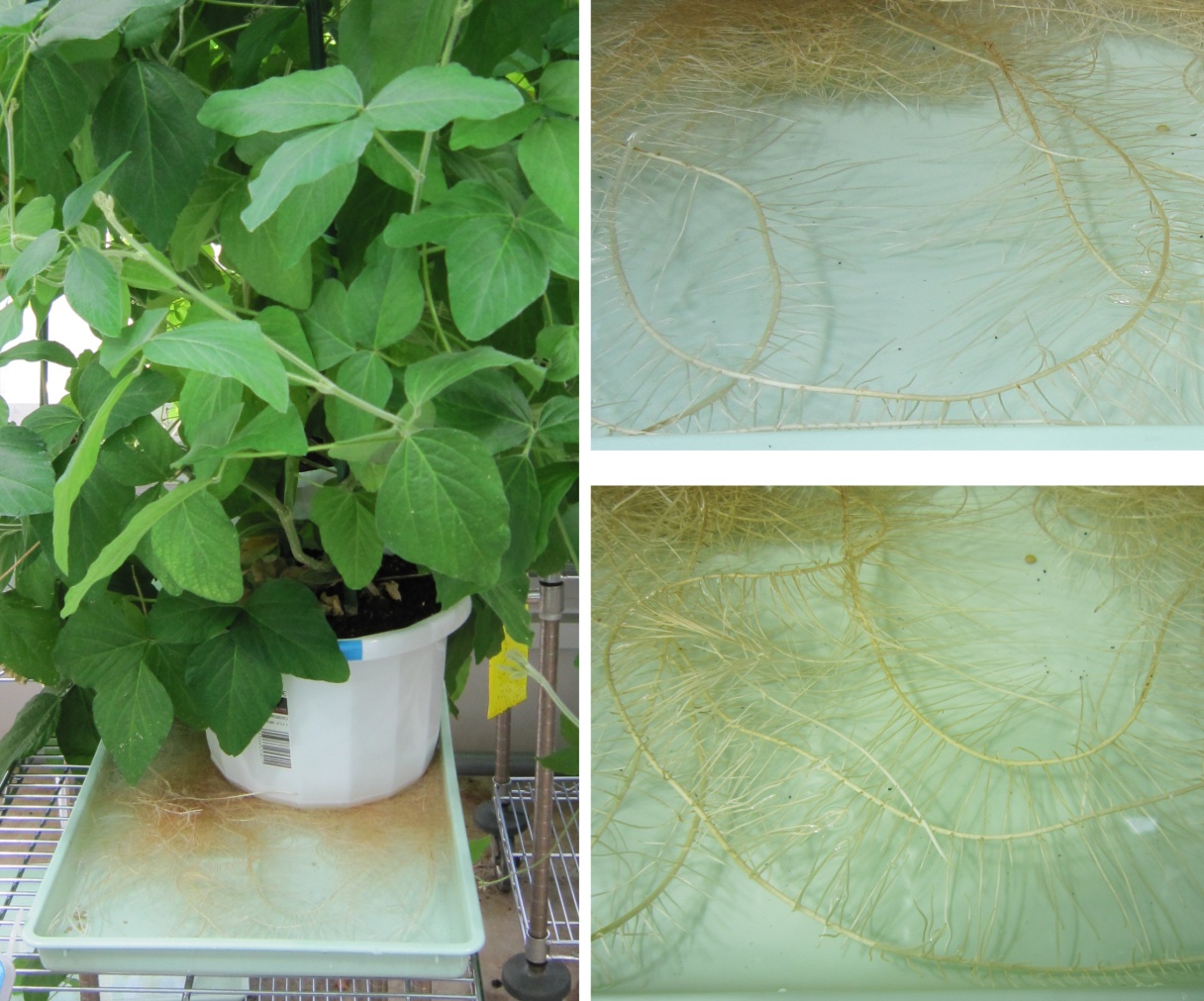


**Figure S2.** **Growth of soybean plants under semi-hydroponic conditions.** Soybean plants were allowed to grow under semi-hydroponic conditions for the collection of root tissues. Detached roots were used for dehydration treatment.
