# Supplementary material for: Identification and Expression Analysis of Cytokinin Metabolic Genes in Soybean under Normal and Drought Conditions in Relation to Cytokinin Levels
Source: PLoS One. 2012 Aug 10;7(8):e42411. doi: 10.1371/journal.pone.0042411 (PMC3416864; doi:10.1371/journal.pone.0042411)
Supplement: Figure S3 — Synteny analysis of GmIPT and GmCKX genes. (A) Synteny analysis revealed evidence of the segmental duplication among several GmIPT genes in soybean. (B) Synteny analysis revealed evidence of the segmental duplication among several GmCKX genes in soybean. (DOC) [file pone.0042411.s003.doc]

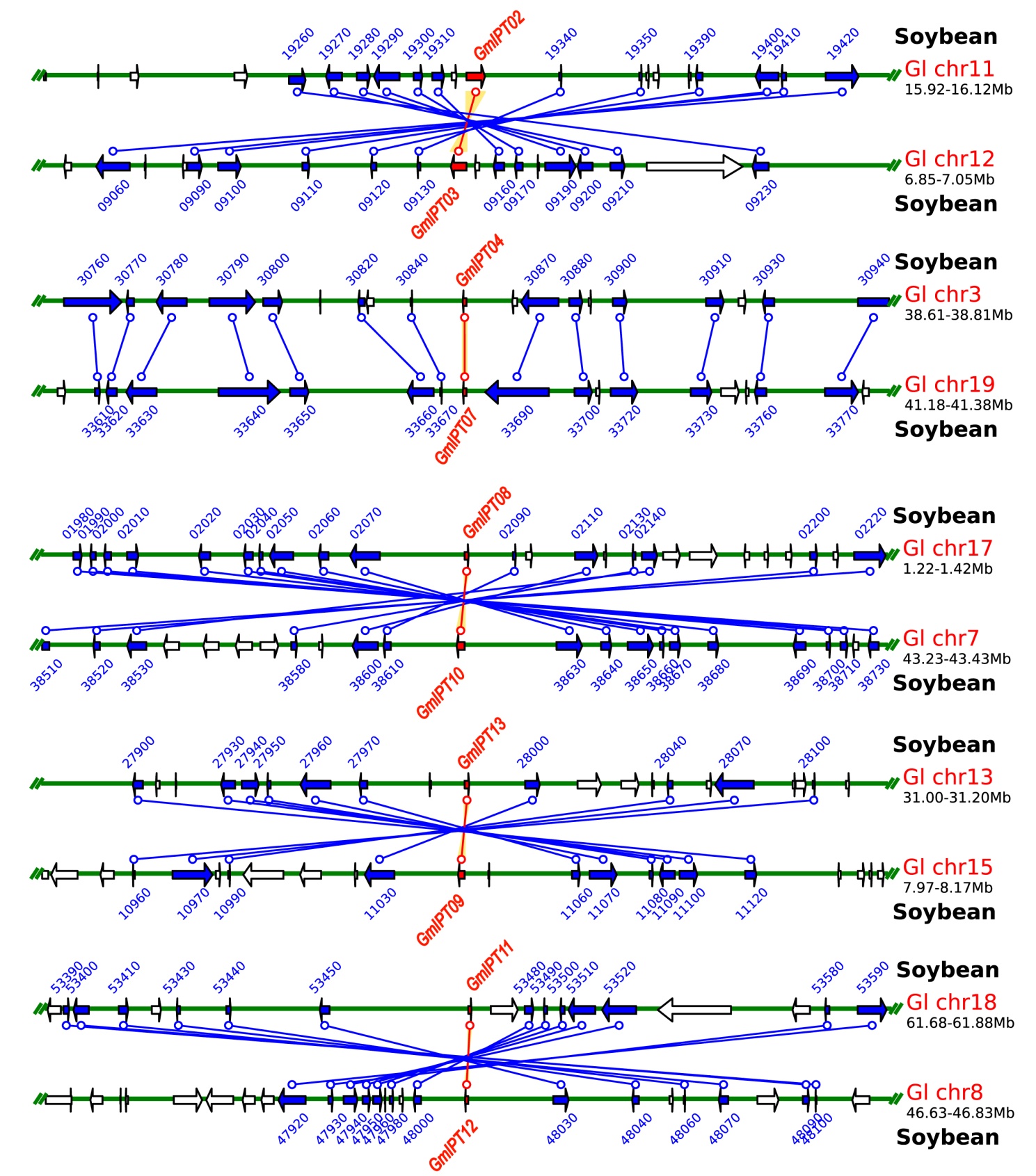


**Figure S3.** **Synteny analysis of *GmIPT* and *GmCKX* genes.** (A) Synteny analysis revealed evidence of the segmental duplication among several *GmIPT* genes in soybean.


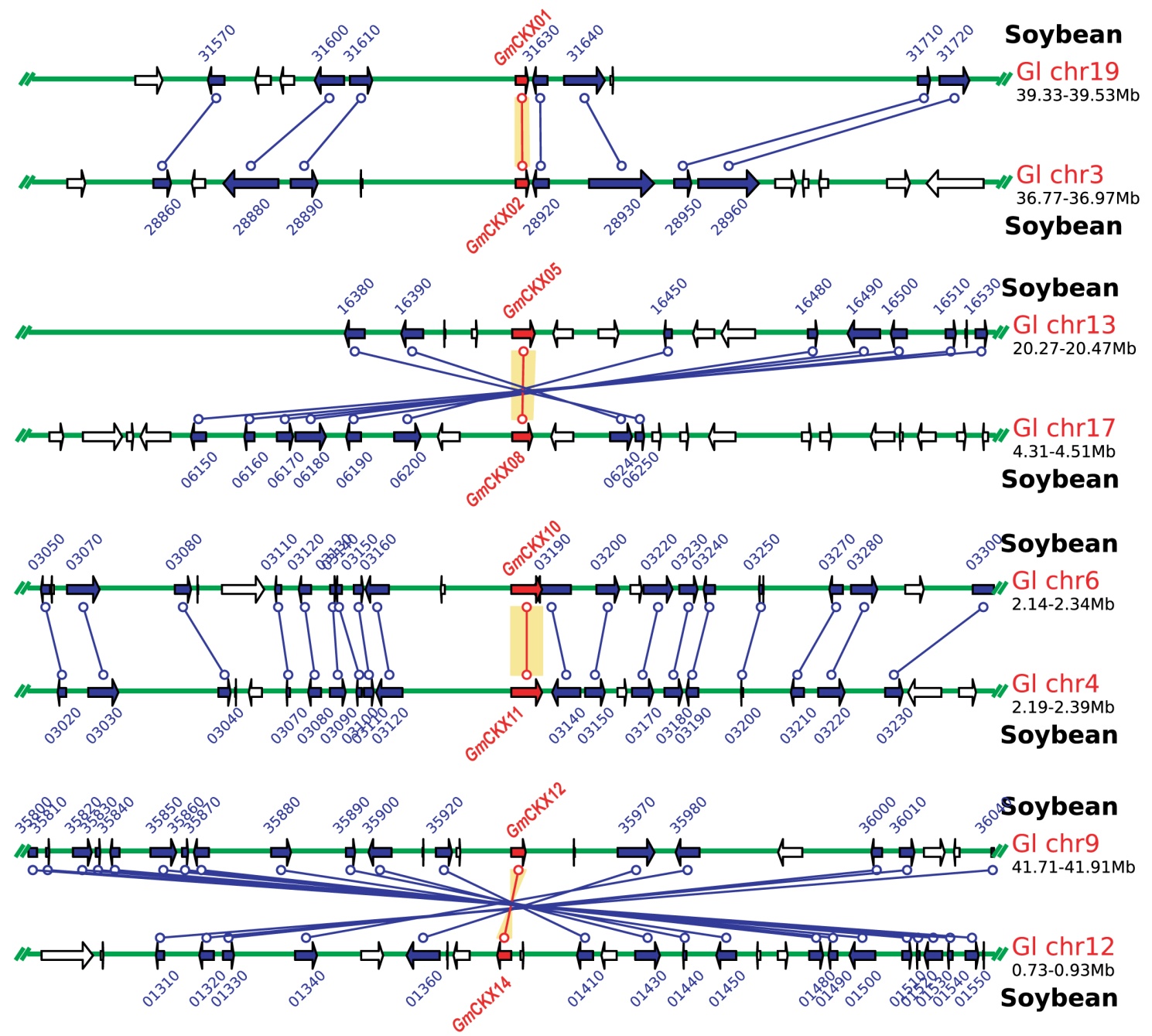


**Figure S3. Synteny analysis of *GmIPT* and *GmCKX* genes.** (B) Synteny analysis revealed evidence of the segmental duplication among several *GmCKX* genes in soybean.
