# Supplementary material for: Identification and Expression Analysis of Cytokinin Metabolic Genes in Soybean under Normal and Drought Conditions in Relation to Cytokinin Levels
Source: PLoS One. 2012 Aug 10;7(8):e42411. doi: 10.1371/journal.pone.0042411 (PMC3416864; doi:10.1371/journal.pone.0042411)
Supplement: Figure S4 — Clustering analysis of tissue-specific expression profiles of GmIPT and GmCKX genes. (A) Expression data (normalized Illumina-Solexa read numbers) collected from Libault et al. (2010) [54]. (B) Expression data from our study. Both two data sets showed that GmIPT02 was highly expressed among the tissues examined and that GmCKX04, 07, 08, 12 and 16 were highly expressed in flowers, suggesting a good agreement between our qRT-PCR data and the data derived from Illumina-Solexa cDNA-sequencing study. (DOC) [file pone.0042411.s004.doc]

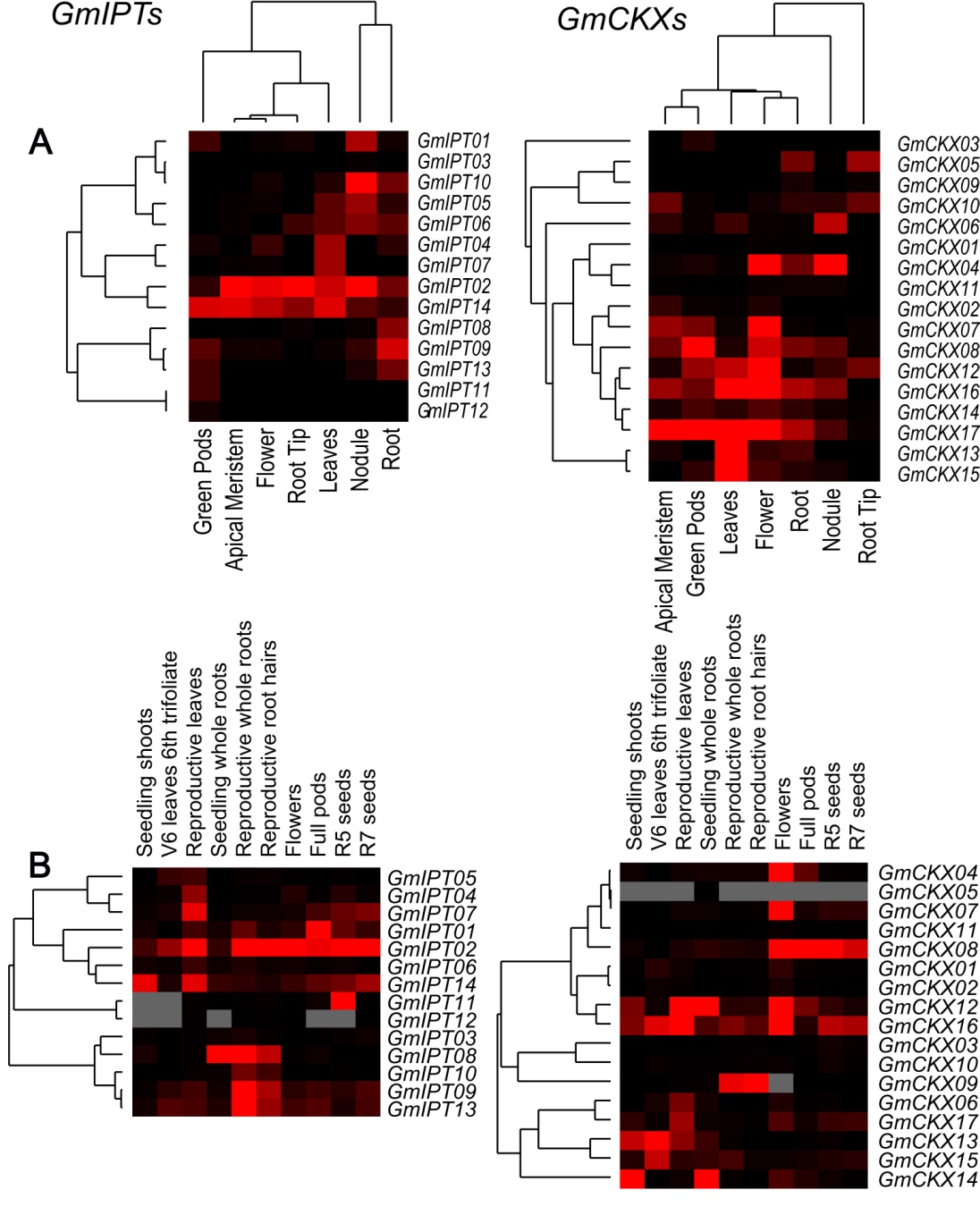


**Figure S4.** Clustering analysis of tissue-specific expression profiles of *GmIPT* and *GmCKX* genes. (A) Expression data (normalized Illumina-Solexa read numbers) collected from Libault et al. (2010) [54]. (B) Expression data from our study. Both two data sets showed that *GmIPT02* was highly expressed among the tissues examined and that *GmCKX04*, *07*, *08*, *12* and *16* were highly expressed in flowers, suggesting a good agreement between our qRT-PCR data and the data derived from Illumina-Solexa cDNA-sequencing study.
