# Supplementary material for: Identification and Expression Analysis of Cytokinin Metabolic Genes in Soybean under Normal and Drought Conditions in Relation to Cytokinin Levels
Source: PLoS One. 2012 Aug 10;7(8):e42411. doi: 10.1371/journal.pone.0042411 (PMC3416864; doi:10.1371/journal.pone.0042411)
Supplement: Table S2 — Primers used for qRT-PCR. (DOC) [file pone.0042411.s006.doc]

**Table S2.** Primers used for qRT-PCR

| **Names** | **Transcript IDs** | **Left** | **Right** |
| --- | --- | --- | --- |
| *GmIPT01* | Glyma10g41990.1 | tccaaaacttcgaccctcac | atatcgacccagaggaagca |
| *GmIPT02* | Glyma11g19330.1 | tttcatgaatgggagcaaca | ccacgaaaccaggaacttgt |
| *GmIPT03* | Glyma12g09140.1 | gccattggtgtccgtgag | gctcccttttctatgctggg |
| *GmIPT04* | Glyma03g30850.1 | gggtgagtttgttgcaggat | ccaccccttaacactcctca |
| *GmIPT05* | Glyma10g03060.1 | tcgcggttcgattttctg | gctcatccaccattccgtta |
| *GmIPT06* | Glyma02g16750.1 | gaggttgctgacgaggagac | tcttccccaactgcttcatc |
| *GmIPT07* | Glyma19g33680.1 | ggtgttgtctctgggtggat | aaagggtctcagctcgttca |
| *GmIPT08* | Glyma17g02080.1 | ttcctctgggtcgatgtttc | aagctttttcgaacctcgtg |
| *GmIPT09* | Glyma15g11040.1 | acttctgtcgctacgccact | ttagagcctccagcgatgat |
| *GmIPT10* | Glyma07g38620.1 | ggcattcctcatcatctgc | catgcgtgtcctcatacagg |
| *GmIPT11* | Glyma18g53460.1 | ctcaatgctctcgatctcatct | tgaggacatgcttgcaaaag |
| *GmIPT12* | Glyma08g48020.1 | tgcaaacatgtcctcaatgc | tttggaatgaaaagtcatgttga |
| *GmIPT13* | Glyma13g27990.1 | tcttcgaccccagtgtaacc | ctctcatcaacgcctccatt |
| *GmIPT14* | Glyma13g34680.1 | gaggatgcaaggcaagctac | aatccagtgcctccaacaac |
| *GmCKX01* | Glyma19g31620.1 | ttgggtcacgatgggaggca | tggccaggggctagaagtgct |
| *GmCKX02* | Glyma03g28910.1 | ggggtttgccgcacagctga | gcttggccttgaagtgaatggcc |
| *GmCKX03* | Glyma09g07190.1 | tcccttgaacgtggactcacacc | acatgccacccatgccagca |
| *GmCKX04* | Glyma09g07360.1 | tacttgacggtgggaggaac | ccagtgatgacgtccatttg |
| *GmCKX05* | Glyma13g16420.1 | caccaacgagggcaaagt | tccagaaaatgcagagaagttg |
| *GmCKX06* | Glyma13g16430.1 | gccacatcgtgcatgaatt | ggggcaaagctattgtagga |
| *GmCKX07* | Glyma15g18560.1 | atctccacagtgggcaaaac | ttgcagaagatggtgtcgtc |
| *GmCKX08* | Glyma17g06220.1 | ggccatttaacgcatgaatt | gctatgtcaaaaggggcaaa |
| *GmCKX09* | Glyma17g06230.1 | aaaatcagccaccactggac | gggaagttatgcgaggttga |
| *GmCKX10* | Glyma06g03180.1 | cgtctcagcgttgacacg | aaggctcccctctactcagc |
| *GmCKX11* | Glyma04g03130.1 | aaggcggcgttcgagtct | cccatttcaatcaccactcc |
| *GmCKX12* | Glyma09g35950.1 | ctgaaatcaattccccttgaag | cagcactgccattggattat |
| *GmCKX13* | Glyma11g20860.1 | aacatttgtcccgcttgaac | tctgaggtatgcaccctgtct |
| *GmCKX14* | Glyma12g01390.1 | gattaagctacatcccgtcga | cgcaacttgacctctgatatg |
| *GmCKX15* | Glyma04g05840.1 | gtcgtcattcagtggtggtg | gtggagtgaatcggagcaag |
| *GmCKX16* | Glyma14g11280.1 | aattggaagtcgtcaccgg | ggtggcgaagaagagttctg |
| *GmCKX17* | Glyma17g34330.1 | ggaattggaggtggtgagc | ggcgaagaagagttccgaat |
