# Supplementary material for: Identification and Expression Analysis of Cytokinin Metabolic Genes in Soybean under Normal and Drought Conditions in Relation to Cytokinin Levels
Source: PLoS One. 2012 Aug 10;7(8):e42411. doi: 10.1371/journal.pone.0042411 (PMC3416864; doi:10.1371/journal.pone.0042411)
Supplement: Table S3 — Drought/dehydration-responsiveness of the soybean and Arabidopsis IPT and CKX genes. (DOC) [file pone.0042411.s007.doc]

**Table S3.** Drought/dehydration-responsiveness of the soybean and *Arabidopsis* *IPT* and *CKX* genes

| Gene name | Chromosome locus | Tissues/regulated by drought/dehydration^a^ | Family^b^ | Regulation by dehydration^c^ |
| --- | --- | --- | --- | --- |
| *GmIPT01* | Glyma10g41990 | Induced in seedling shoots and R2 root hairs | *AtIPT1/AtIPT8/AtIPT6* | Repressed |
| *GmIPT02* | Glyma11g19330 | Induced in young trifolia, repressed in aged trifolia | *AtIPT2* | No data |
| *GmIPT03* | Glyma12g09140 | Induced in seedling shoots , roots, young trifolia and root hairs | *AtIPT2* | No data |
| *GmIPT04* | Glyma03g30850 | Induced in seedling roots and shoots | *AtIPT3* | Repressed |
| *GmIPT05* | Glyma10g03060 | Repressed in seedling shoots, leaves but induced in root hairs | *AtIPT3* | Repressed |
| *GmIPT06* | Glyma02g16750 | Repressed in seedling shoots, leaves | *AtIPT3* | Repressed |
| *GmIPT07* | Glyma19g33680 | Induced in seedling roots, shoots, root hairs, but repressed in R2 leaves | *AtIPT3* | Repressed |
| *GmIPT08* | Glyma17g02080 | Induced in seedling shoots, V6 and R2 leaves and R2 root hairs | *AtIPT5* | Slightly induced |
| *GmIPT09* | Glyma15g11040 | Induced in shoots, young trifolia and R2 root hairs, repressed in seedling roots | *AtIPT5* | Slightly induced |
| *GmIPT10* | Glyma07g38620 | Repressed in seedling roots, trifolia leaves, induced in seedling shoots | *AtIPT5* | Slightly induced |
| *GmIPT11* | Glyma18g53460 | Induced in seedling roots, shoots and R2 root hairs | *AtIPT5/AtIPT7* | *AtIPT7* repressed |
| *GmIPT12* | Glyma08g48020 | Induced in seedling roots, shoots and R2 root hairs | *AtIPT5/AtIPT7* | *AtIPT7* repressed |
| *GmIPT13* | Glyma13g27990 | Induced in seedling shoots, trifolia leaves, R2 root hairs, repressed in seedling roots | *AtIPT5* | Slightly induced |
| *GmIPT14* | Glyma13g34680 | Complex response | *AtIPT9* | No data |
| *GmCKX01* | Glyma19g31620 | Repressed in leaves | *AtCKX1/AtCKX6* | Repressed |
| *GmCKX02* | Glyma03g28910 | Repressed in leaves | *AtCKX1/AtCKX6* | Repressed |
| *GmCKX03* | Glyma09g07190 | Induced in roots, shoots and leaves | *AtCKX2* | Repressed |
| *GmCKX04* | Glyma09g07360 | Induced in roots, shoots and leaves | *AtCKX3* | Repressed |
| *GmCKX05* | Glyma13g16420 | expression too low | *AtCKX3* | Repressed |
| *GmCKX06* | Glyma13g16430 | Repressed in seedling roots, shoots, leaves, induced in R2 roots | *AtCKX3* | Repressed |
| *GmCKX07* | Glyma15g18560 | V6 leaves, induced | *AtCKX3* | Repressed |
| *GmCKX08* | Glyma17g06220 | Induced in roots, repressed in shoots | *AtCKX3* | Repressed |
| *GmCKX09* | Glyma17g06230 | Roots seedlings, V6 leaves /repressed; R2 root hairs/induced | *AtCKX3/AtCKX4* | Repressed |
| *GmCKX10* | Glyma06g03180 | Induced in roots or root hairs only | *AtCKX5* | Repressed |
| *GmCKX11* | Glyma04g03130 | roots, leaves / induced in roots or root hairs only | *AtCKX5* | Repressed |
| *GmCKX12* | Glyma09g35950 | Repressed in R2 leaves, induced in R2 roots/root hair | *AtCKX6* | Slightly repressed |
| *GmCKX13* | Glyma11g20860 | Repressed in shoots, leaves; induced in R2 roots | *AtCKX6* | Slightly repressed |
| *GmCKX14* | Glyma12g01390 | Induced in shoots, leaves and R2 roots/root hair | *AtCKX6* | Slightly repressed |
| *GmCKX15* | Glyma04g05840 | Repressed in roots, shoots, leaves | *AtCKX7* | Repressed |
| *GmCKX16* | Glyma14g11280 | Repressed in roots | *AtCKX7* | Repressed |
| *GmCKX17* | Glyma17g34330 | Repressed in roots, shoots of seedlings and R2 leaves | *AtCKX7* | Repressed |

^a^Drought/dehydration-responsiveness of *GmIPT* and *GmCKX* genes from our study

^b^According to the closest *Arabidopsis* orthologous gene(s) shown in Tables 1and 2

^c^Dehydration-responsiveness of *AtIPT* and *AtCKX* genes in 10-d-old *Arabidopsis* seedlings. Data derived from reference [11]
