# Supplementary material for: Identification and Expression Analysis of Cytokinin Metabolic Genes in Soybean under Normal and Drought Conditions in Relation to Cytokinin Levels
Source: PLoS One. 2012 Aug 10;7(8):e42411. doi: 10.1371/journal.pone.0042411 (PMC3416864; doi:10.1371/journal.pone.0042411)
Supplement: Table S4 — CK contents in various soybean tissues under normal and drought stress conditions. (A) Concentration of individual CK metabolites in various soybean tissues. (B) CK contents in various soybean tissues in group of compounds. (DOC) [file pone.0042411.s008.doc]

**Table S4. CK contents in various soybean tissues under normal and drought stress conditions.** (A) Concentration of individual CK metablolites in various soybean tissues

|  | *tZ* | *tZ-sd* | *tZR* | *tZR-sd* | *tZOG* | *tZOG-sd* | *tZROG* | *tZROG-sd* | *tZRMP* | *tZRMP-sd* | *DZ* | *DZ-sd* | *DZR* | *DZR-sd* |
| --- | --- | --- | --- | --- | --- | --- | --- | --- | --- | --- | --- | --- | --- | --- |
| **Leaves** | 1.31 | 0.11 | N.D. | - | 3.73 | 0.52 | N.D. | - | N.D. | - | 3.87 | 1.29 | 0.83 | 0.56 |
| **Leaves - drought** | 0.67 | 0.46 | N.D. | - | 4.34 | 0.75 | N.D. | - | N.D. | - | 1.30 | 0.17 | 0.61 | 0.34 |
| **Young leaves** | 2.09 | 0.63 | 0.52 | 0.30 | 0.90 | 0.12 | N.D. | - | N.D. | - | 2.15 | 1.34 | 0.54 | 0.18 |
| **Whole roots** | N.D. | - | N.D. | - | N.D. | - | N.D. | - | 5.61 | 1.38 | 2.45 | 0.41 | 1.25 | 0.42 |
| **Root tips** | 2.33 | 0.24 | N.D. | - | 0.08 | 0.17 | N.D. | - | 6.39 | 2.89 | 1.53 | 0.18 | 2.13 | 0.16 |
| **Root hairs** | 0.40 | 0.35 | N.D. | - | 0.04 | 0.07 | N.D. | - | 10.1 | 2.91 | 0.29 | 0.06 | 1.20 | 0.58 |
| **Flowers** | 1.50 | 0.30 | N.D. | - | 1.09 | 0.40 | N.D. | - | 1.43 | 0.43 | 2.39 | 0.66 | 0.85 | 0.55 |
| **Full pods** | 17.6 | 2.35 | 80.6 | 4.07 | 3.96 | 0.21 | 4.44 | 1.57 | 18.1 | 1.48 | 5.56 | 0.18 | 23.4 | 2.63 |
|  | *DZOG* | *DZOG-sd* | *DZRMP* | *DZRMP-sd* | *iP* | *iP-sd* | *iPR* | *iPR-sd* | *iPRMP* | *iPRMP-sd* | *cZ* | *cZ-sd* | *cZOG* | *cZOG-sd* |
| **Leaves** | 1.43 | 0.19 | N.D. | - | 0.11 | 0.03 | 2.64 | 0.82 | 1.50 | 0.74 | N.D. | - | 1.85 | 0.72 |
| **Leaves - drought** | 1.81 | 0.66 | N.D. | - | N.D. | - | 2.71 | 0.31 | 2.07 | 0.42 | N.D. | - | 1.17 | 0.61 |
| **Young leaves** | N.D. | - | 20.8 | 5.61 | 0.37 | - | 0.48 | 0.21 | 2.33 | 0.90 | 0.06 | 0.04 | 0.56 | 0.39 |
| **Whole roots** | N.D. | - | N.D. | - | 0.56 | 0.07 | 0.41 | 0.12 | 3.84 | 2.06 | 0.55 | 0.29 | N.D. | - |
| **Root tips** | N.D. | - | 3.61 | - | 0.58 | 0.23 | 0.72 | 0.39 | 9.21 | 1.87 | N.D. | - | N.D. | - |
| **Root hairs** | N.D. | - | N.D. | N.D. | 0.60 | 0.29 | 0.29 | 0.18 | 8.39 | 0.76 | 0.21 | - | 0.23 | 0.15 |
| **Flowers** | N.D. | - | 3.33 | 0.62 | N.D. | - | 0.57 | 0.24 | 0.66 | 0.12 | N.D. | - | N.D. | - |
| **Full pods** | 0.9 | 0.08 | 5.62 | 2.12 | 1.14 | - | 4.38 | 0.57 | 2.94 | 0.49 | N.D. | - | N.D. | - |

100 milligrams of tissues per sample were collected. Three independent biological samples were taken for each tissue/treatment.

Data shown are pmol/g fresh weight (n = 3). For “Leaves” and “Leaves-drought” samples, the leaf RWCs were 91 ± 1% and 32 ± 2%, respectively. *t*Z, *trans*-zeatin; *t*ZR, *t*Z riboside; *t*ZRMPs, *t*ZR 5'-monophosphates; *c*Z, *cis*-zeatin; DZ, dihydrozeatin; DZR, DZ riboside; DZRMPs, DZR 5'-monophosphates; iP, N6-(Δ^2^-isopentenyl) adenine; iPR, iP riboside; iPRMPs, iPR 5'-monophosphates; *t*ZOG, *t*Z-*O*-glucoside; *c*ZOG, *c*Z-*O*-glucoside; N.D., not detectable.

|  |
| --- |
|  |
|  |

**Table S4.** **CK contents in various soybean tissues under normal and drought stress conditions.** (B) CK contents in various soybean tissues in group of compounds

|  | *tZ* | *tZR+tZRPs* | *tZOG+tZROG* | *DZ* | *DZR+DZRPs* | *iP* | *iPR+iPRPs* | *cZ* | *cZOG* | *sum all* |
| --- | --- | --- | --- | --- | --- | --- | --- | --- | --- | --- |
| **Leaves** | 1.31 | N.D. | 3.73 | 3.87 | 0.83 | 0.11 | 4.14 | N.D. | 1.85 | 15.83 |
| **leaves - drought** | 0.67 | N.D. | 4.34 | 1.30 | 0.61 | N.D. | 4.78 | N.D. | 1.17 | 12.86 |
| **Young leaves** | 2.09 | 0.52 | 0.90 | 2.15 | 21.33 | 0.37 | 2.81 | 0.06 | 0.56 | 30.78 |
| **Whole roots** | N.D. | 5.61 | N.D. | 2.45 | 1.25 | 0.56 | 4.26 | 0.55 | N.D. | 14.68 |
| **Root tips** | 2.33 | 6.39 | 0.08 | 1.53 | 5.74 | 0.58 | 9.93 | N.D. | N.D. | 26.59 |
| **Root hairs** | 0.40 | 10.06 | 0.04 | 0.29 | 1.20 | 0.60 | 8.68 | 0.21 | 0.23 | 21.72 |
| **Flowers** | 1.50 | 1.43 | 1.09 | 2.39 | 4.18 | N.D. | 1.23 | N.D. | N.D. | 11.81 |
| **Full pods** | 17.63 | 98.75 | 8.40 | 5.56 | 29.02 | 1.14 | 7.32 | N.D. | N.D. | 167.83 |
